# Improving Quality Metrics in Neurosurgery: A Spinal Surgery 3-Year Case Review

**DOI:** 10.1227/neuprac.0000000000000071

**Published:** 2024-01-30

**Authors:** Christina Nicoll Feller, Erin M. Bodenbach, Julie M. Kolinski, Grant P. Sinson

**Affiliations:** * Medical College of Wisconsin, Milwaukee, Wisconsin, USA;; ‡ Department of Medicine, Medical College of Wisconsin, Milwaukee, Wisconsin, USA;; § Department of Neurosurgery, Medical College of Wisconsin, Milwaukee, Wisconsin, USA

**Keywords:** Clinical documentation, DRG, Neurosurgery, Quality improvement, Spinal surgery

## Abstract

**BACKGROUND AND OBJECTIVES:**

Despite the known importance of accurate clinical documentation as a companion to quality patient care, this is not often prioritized in practice and leads to a variety of downstream consequences. Inaccurate documentation leads to missed opportunities in full, accurate coding. In turn, it also negatively influences hospital and physician quality ranking, medical center profiling, and revenue captured. The aim of this study is to highlight the opportunity for continuous improvement in clinical documentation and the significance accurate clinical documentation has on outcome measures, such as expected length of stay (eLOS).

**METHODS:**

A single-center retrospective chart review took place for patients undergoing spinal surgery from 2019 to 2021. Based on Vizient's diagnosis-related group risk model for eLOS, 192 charts spanning 10 unique diagnosis-related groups were reviewed to identify ICD-10 diagnosis and procedure codes that were not coded or not clearly documented by a physician. A new eLOS for each patient was recalculated with the addition of the newly identified variables and then compared with the original eLOS.

**RESULTS:**

Overall, there was a significant difference between the original eLOS and new eLOS when the newly identified variables were added (*P* < .001). Of 192 patient charts, 89.5% had at least one new variable contributing to eLOS, with an average of 2.60 (0, 12) new variables. This resulted in an average increase in eLOS of 2.869 days (−0.160, 35.129). Compared with the observed LOS, the new eLOS was significantly different (*P* < .001), whereas the original LOS was not (*P* = .5661).

**CONCLUSION:**

Incomplete documentation and coding can misrepresent the quality of patient care provided and the complexity of their cases. This represents an opportunity for improvement for both the clinicians, clinical documentation improvement specialists, and coders to improve quality metrics and hospital rankings.

**ABBREVIATIONS:**
CC: complication or comorbidity
CDB: Clinical Data Base
DRG: diagnosis-related group
eLOS: expected length of stay
MCC: major complication or comorbidity
oLOS: observed length of stay.

Documentation of care delivered to surgical patients is a universally important aspect of medicine. Clinical documentation of all patients, including spinal surgeries, affects the coding accuracy and validity of Diagnosis-Related Groups (DRGs). Both patient complication or comorbidity (CC) and major complication or comorbidity (MCC) play into a final Case Mix Index. Case Mix Index affects physician and medical center profiling, quality reporting, and revenue captured. By targeting upstream and improving clinical documentation, a positive downstream effect can provide a more accurate representation of hospital care and increase hospital quality metrics.

However, improving clinical documentation can prove to be challenging. Clinicians may not see the importance of documenting with coding in mind. It is difficult to remember and note the specific variables coders are looking for in each patient population. Documentation is primarily a way for physicians and advanced practice providers to communicate with each other, not with coders. Thus, another difficulty arises in the coding process—interpretation of clinical documentation by coders. Coders might have a difficult time in interpreting medical jargon commonly used for specific diagnoses prohibiting the coding of those diagnoses appropriately because of coding rules. Both factors have a detrimental effect on quality rankings for the provider and hospital because the quality of care is seemingly decreased while, it is merely inaccurate back-end processing.

In this study, we aimed to examine the Vizient Clinical Data Base (CDB) outcome metric, expected length of stay (eLOS). In the United States, Vizient's services are used by approximately 97% of the nation's academic medical centers, 60% of acute care hospitals, and greater than 25% of the nonacute market. Their services and analytics are used to benchmark care and identify opportunities to change care delivery, which ultimately improves metrics. It has been previously shown that eLOS can be greatly affected by high-acuity departments such as the department of neurosurgery.^1^ LOS is a particularly useful metric because it is relatable to clinicians and is often trended by healthcare systems. It is also the most applicable in this setting because spine procedures have a low mortality rate and cost metrics are incredibly variable and dependent on the method used. Furthermore, previous research studies have found that by addressing and enhancing clinical documentation, quality metrics and rankings increase positively within varying neurosurgical departments.^2,3^ This study aims to analyze documentation within our own Department of Neurosurgery over the course of 3 years to identify trends and opportunities for more accurate documentation and its significance on influencing outcome measures, such as eLOS.

## METHODS

The primary resource for data collection was through the Vizient CDB. Vizient is a healthcare performance company that serves as a quality-of-care comparison cohort among approximately 95% of academic medical centers and over 300 community hospitals. This project was undertaken as a quality improvement initiative and did not include any interaction or intervention with human subjects or patient identifiers. Therefore, Institutional Review Board/ethics committee approval and patient consent were neither needed nor obtained.

For all spinal surgeries occurring in one academic center, the Vizient CDB was used to analyze the DRGs corresponding to spinal surgeries in 2019 (6 DRGs analyzed), 2020 (5), and 2021 (6).

Across the 3 years, retrospective chart review took place based on Vizient CDB's DRG risk models for eLOS. One hundred and ninety-two charts spanning 10 unique DRGs were reviewed to identify 10th revision of the International Statistical Classification of Diseases and Related Health Problems (ICD-10) diagnosis and procedure codes that were not coded or not clearly documented. These missed variables for eLOS were recorded as potential points to increase accuracy of clinical documentation. Expected LOS was then recalculated with the addition of previously missed variables discovered during chart review, thus creating a new eLOS for each patient. The most missed variables were gathered to determine points for improvement in future years. This method was repeated for spinal surgery cases that occurred in 2020 and 2021. Data were compared to analyze trends and opportunities in the accuracy of clinical documentation and coding of LOS variables.

## RESULTS

Over all 3 years, the most missed variables were those highlighting obesity, physical condition factors including fatigue or weakness, and electrolyte imbalances including issues with potassium, magnesium, or acid–base disturbances. From January 2019 to October 2019, 76 total spinal surgery cases were reviewed with an average eLOS of 4.48 days. Sixteen different surgeons performed these cases. Scores are based on five different domains: mortality, efficiency, safety, effectiveness, and patient centeredness. From chart reviews of spinal procedures performed in 2019, at least one new variable LOS was found in 85.5% of cases with an average of one new variable found. After including these variables, the eLOS was recalculated with an average increase of 1 day (0-7 days) (Table).

**TABLE.** Data Summary Across 3 Years

Data Calculated	2019 (N = 76)	2020 (N = 38)	2021 (N = 78)
Average number of new variables found per patient	1.24 (0, 5)	4.24 (0, 12)	3.13 (0, 8)
Percent of charts with 1+ new variables	85.5% (65)	92.1% (35)	92.3% (72)
Average difference between original eLOS and recalculated eLOS	+0.725 days (−0.16, 6.569)	+6.87 days (0, 35.13)	+2.883 days (0, 22.789)

eLOS, expected length of stay.

From July 2020 to August 2020, a total of 38 spinal surgeries by 10 different surgeons were reviewed, with an average of four new variables found per chart (0-12). Ninety-two percent of the charts (N = 35) contained at least one or more new variables, which encoded for an average increase of 7 days (0-35 days). From January of 2021 to August 2021, a total of 78 spinal surgery patient charts were reviewed whose cases were performed by 18 different surgeons, with an average of three new variables found per chart (0-8). Ninety-two percent of these charts had one or more new variable found, causing an average increase in LOS of 3 days (0-23 days) (Table).

**FIGURE 1.**
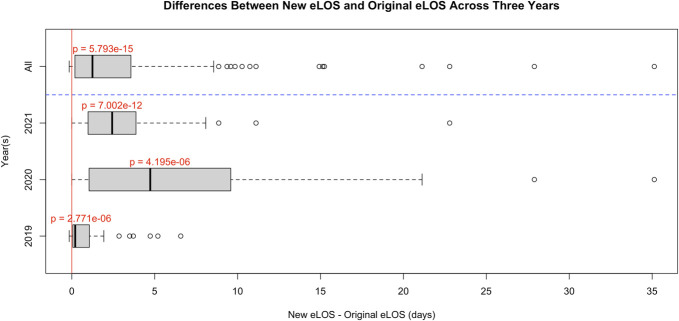
Differences between new eLOS and original eLOS across 3 years. A paired *t*-test was conducted to compare the estimated length of stay calculated by the Vizient Clinical Data Base before the addition of new explanatory variables identified in chart review and after their addition. eLOS, expected length of stay.

When comparing Vizient CDB's initial predicted length of stay with the recalculated length of stay including missed variables, there was an overall significant increase in the mean eLOS once the omitted variables were included (t(191) = 8.4876, *P* < .001). This held true when analyzing each year independently (2019: t(75) = 5.0713, *P* < .001; 2020: t(37) = 5.3909, *P* < .001; 2021: t(77) = 8.0842, *P* < .001) (Figure 1).

**FIGURE 2.**
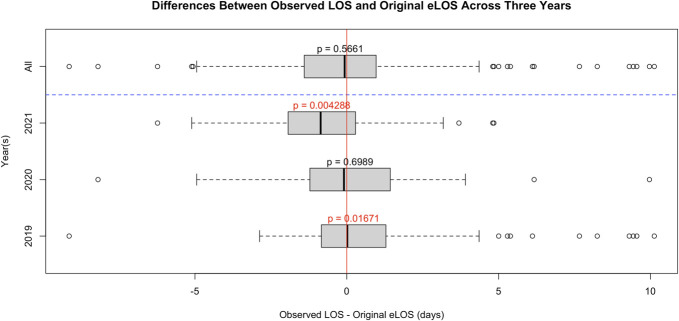
Differences between observed LOS and original eLOS across 3 years. A paired *t*-test was conducted to compare the observed length of stay with the estimated length of stay calculated by the Vizient Clinical Data Base without the addition of new explanatory variables identified in chart review. eLOS, expected length of stay.

A paired *t*-test was conducted between Vizient CDB's eLOS without including new variables, and the actual observed LOS (oLOS) demonstrated no significant difference (t(191) = 0.57479, *P* = .5661). When grouped by year, there is still no significant difference for 2020 (t(37) = 0.38978, *P* = .6989), but there was a significant difference between the expected and oLOS for 2019 and 2021 (2019: t(75) = 2.4478, *P* < .02; 2021: t(77) = −2.9435, *P* < .01) (Figure 2).

**FIGURE 3.**
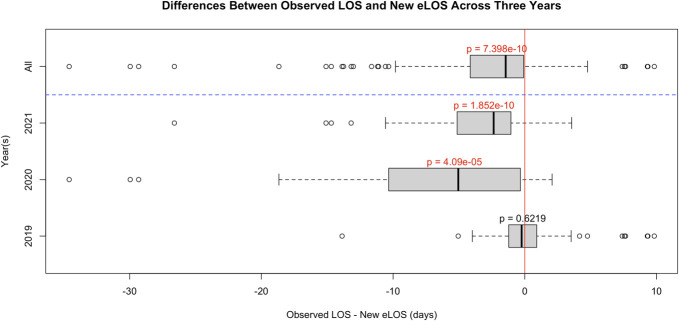
Differences between observed LOS and new eLOS across 3 years. A paired *t*-test was conducted to compare the observed length of stay with the estimated length of stay when the new explanatory variables identified in chart review were accounted for. eLOS, expected length of stay.

On the other hand, when comparing Vizient CDB's eLOS with including new variables to the oLOS was significantly shorter than the recalculated LOS (t(191) = −6.4851, *P* < .001). When grouped by year, there was a significant difference for 2020 and 2021 (2020: t(37) = −4.6537, *P* < .001; 2021: t(77) = −7.3429, *P* < .001), but not for 2019 (t(75) = 0.49527, *P* = .6219) (Figure 3).

## DISCUSSION

It is well accepted that improved documentation leads to enhanced quality metrics through accurate capture of severity of illness of patient populations and their comorbid conditions; however, what is not well known is the degree of this enhancement on the deficits created by poor documentation. Our data demonstrate consistent opportunity for improvement of clinical documentation over 3 years. While there was a significant increase in all metrics from 2019 to 2020 (average number of new variables found per chart, percent of charts with at least one new variable found, and average change in eLOS), there was a slight downtrend from 2020 to 2021. This, in part, may be due to the slowing of cases during the beginning of the COVID-19 pandemic and the smaller sample size within the 2020 data set. In addition, during this timeframe, efforts were underway to improve variable capture by our Clinical Documentation specialists in our hospital system's Clinical Documentation Improvement (CDI) program. However, this initiative is a lengthy project to fully implement. In addition to CDI, technology-based solutions have been used to make providers more aware of the impact of their documentation.^4^ Overall, these data show that there is still room for great improvement within clinical documentation and the vast potential accurate documentation has on coding, hospital quality ranking, and potential hospital reimbursement. Providers at other institutions can use these data and others like these to ask their administrative teams if efforts to capture all these variables in their patients have been maximized.

Furthermore, while the oLOS was not significantly different from the original eLOS, it was significantly different from the new, recalculated eLOS. This demonstrates that because the Vizient CDB is built on historical data and coding, the model is accurate when compared with oLOS. However, the data show a decreased accuracy of outcomes when missing or omitted variables are considered. As coding accuracy universally improves over time, Vizient CDB's predicted length of stay will more accurately predict LOS while also maintaining a high standard of comprehensive coding.

### Documentation

Accurate documentation of patients is critical in accurate coding, hospital ranking, and reimbursement.^5^ Providers are tasked to document all patient CCs and MCCs that are essential for each case. Each patient encounter is associated with a certain diagnostic-related group (DRG), which, in turn, has its own set of variables. These variables are the factors determining the documented eLOS for the specific procedure. For example, if Patient A comes in with no CCs or MCCs, their eLOS will be lower than Patient B who has significant CCs and MCCs that increase the number of variables able to be coded. Because a patient accrues more variables, the expectation is that it is more difficult to care for such a patient, and thus, the eLOS increases. If a hospital/provider exceeds expectations (discharges before the eLOS), the assumption is that this hospital/provider provided excellent care, thus increasing the hospital's quality ranking, provider's quality ranking, and reimbursement.

Quality patient care and documentation relies heavily on the collaboration between physicians and the other members of the team, including nursing, physical and occupational therapy, and social work. These individuals often contribute essential information to a patient's chart with their own assessments and plans. Of note, weights are typically recorded by nursing staff, and physical and occupational therapists detail the patient's mobility and functionality. However, this documentation is not what will be used by coders. When the documentation needed for coding falls onto the provider, it may seem frivolous to repeat information provided by other members of the healthcare team in their own documentation such as obesity or reduced mobility.

With increased patient access to their own medical record, the line between words that express a medical diagnosis or description and its cultural connotations is often blurred. For instance, while describing a patient as “obese” is a variable that is commonly used to determine eLOS and can be an indicator of overall patient health, it may be viewed by a patient as a negative statement and may even place strain on the patient–physician relationship.

Overall, the most missed variables in documentation are nutritional factors such as “fluid & electrolyte disorders,” “nutritional anemia,” and “vitamin and mineral deficiencies.”^6^ This correlates with our results in that the third most missed variable was due to electrolyte imbalances including issues with potassium, magnesium, or acid–base disturbances. Furthermore, decreased iron levels led to our fourth most missed variable. Because laboratory values autopopulate in the results of a patient chart, it can become more of an action item to address clinically rather than something a physician feels the need to document. However, this lack of explicit documentation means that it cannot be coded and another variable is easily missed.

### Coding

While improvements in clinical documentation can increase hospital quality metrics and rankings, accurate coding can also positively influence Vizient metrics. Accurate coding relies heavily on clinical documentation being accurately translated and all diagnoses being present in the medical record to be coded. However, the language used by clinicians differs significantly from than that used by coders.^2^ Providers are reliant on medical knowledge and language to communicate between healthcare workers in the most accurate yet quickest manner. Thus, many providers omit certain information that may not seem as relevant to the current admission to facilitate quick and easy communication between care teams. However, they may be unaware of the downstream effect this has on coding, which, in turn, affects reimbursement and their own and their hospital's quality ranking. These condensed notes providers’ document may not only omit certain information but also contain medical jargon that coders may not be able to use because of coding guidelines. Differences in language between medical providers and coders create a barrier to accurate clinical documentation and the coding process. A discrepancy in translation may lead to missed variables, CCs, and MCCs.

### Limitations and Future Steps

One limitation to our study is the fact that only data from the neurosurgical department in one academic center were collected. Generalizability may be limited because hospital structure, types of procedures commonly performed, and individual familiarity with the coding process all vary both within hospitals' individual departments and across different hospitals. Furthermore, the risk model calculator used to determine eLOS and the researcher collecting variables changed between the years of 2019 and 2021. Vizient updated their DRG risk models for eLOS in 2021, and with that, the number and impact of the variables changed in addition to alterations in the variables themselves. Another limitation is the changing DRGs between years. In 2019, DRGs 13, 146, 147, 148, 153, and 160 were analyzed, whereas in 2020, DRGs 146, 171, 177, 180, and 328 were analyzed. Finally, DRGs analyzed for 2021 cases included 147, 153, 155, 160, 162, and 180. Besides Vizient's DRG risk models changing, another limitation is the range of data points collected per year (ranging from 38 to 78). The drop in cases analyzed in 2020 can in part be explained by the COVID-19 pandemic with a slowing of cases in 2020. In addition, during this timeframe, efforts were underway to improve variable capture by our Clinical Documentation specialists, potentially leaving few variables uncoded.

Future steps involve creating educational programs for both providers and coders to bridge the gap in language and communication and highlight the importance of complete and accurate documentation in hospital quality rankings. Another avenue to explore is how the quality of documentation varies between the different procedures performed in the neurosurgical department or if certain patient populations are associated with more accurate and complete coding than others. Continuous research into the evolution and consistent need for quality of documentation across the years should be pursued as hospitals continue to evolve and resume to work at a normal case load.

## CONCLUSION

This study highlights that there appear consistent opportunity and potential for more accurate clinical documentation. Improved clinical documentation leads to a more accurate representation of care being implemented for a patient undergoing spinal surgery and furthermore increased hospital quality metrics. On the other hand, inadequate clinical documentation of surgeries alters the coding accuracy and validity of DRGs and associated patient CCs or MCCs, in turn, leading to misrepresentation of the quality of patient care being provided and a quality ranking below its potential. Ensuring that providers continue to improve their documentation skills over the course of their practice will not only benefit individual physician profiling and medical center profiling but also increase revenue captured and lead to a more accurate representation of hospital care.
